# Effects of Poly-γ-Glutamic Acid Molecular Weight on Lettuce Growth, Soil Properties, and Bacterial Community Structure

**DOI:** 10.3390/polym18050640

**Published:** 2026-03-05

**Authors:** Yu Lin, Linye Wang, Lin Shu, Huizhen Chen, Zhiqun Liang, Wei Zeng

**Affiliations:** 1 School of Basic Medicine Sciences, Guilin Medical University, Guilin 541199, China; linyu2023@glmu.edu.cn (Y.L.); sl27580493@126.com (L.S.); 222023507@glmu.edu.cn (H.C.); 2 State Key Laboratory for Conservation and Utilization of Subtropical Agro-Bioresources, Guangxi Microorganism and Enzyme Research Center of Engineering Technology, College of Life Science and Technology, Guangxi University, Nanning 530004, China; 19077306961@163.com (L.W.); zw4628593@163.com (Z.L.); 3 School of Biomedical Industry, Guilin Medical University, Guilin 541199, China

**Keywords:** poly-γ-glutamic acid, molecular weight, soil water-holding capacity, bacterial community structure, lettuce growth

## Abstract

Poly-γ-glutamic acid (γ-PGA) can regulate soil physicochemical properties and enhance crop yield. However, the effect of γ-PGA molecular weight (Mw) on plant growth remains unclear. In this study, we investigated the effects of γ-PGAs with low (70–100 kDa), high (700–1100 kDa), and ultra-high (>3000 kDa) Mws on lettuce growth and soil properties. The results showed that γ-PGA application reduced the infiltration rate of red soil. In pot experiments, γ-PGAs with different Mws at 0.1% promoted lettuce growth, and blade length and width increased with increasing Mw. However, the excessive application of ultra-high Mw γ-PGA inhibited lettuce growth. Soil chemical properties revealed that γ-PGA treatments significantly increased soil ammonium nitrogen and available potassium content. Furthermore, bacterial community structure analysis indicated that adding γ-PGA reduced bacterial diversity and richness, particularly under low and high Mw γ-PGA treatments, while increasing the relative abundance of beneficial plant-associated bacteria, including *Proteobacteria* and *Acidobacteriota*. Overall, ultra-high Mw γ-PGA exhibited the strongest effects on soil water retention and nutrient regulation, whereas low application rate was more favorable for plant growth. These findings can provide insights into the agricultural application of γ-PGA.

## 1. Introduction

Poly-γ-glutamic acid (γ-PGA) is a natural polymer derived from microorganisms through an amide bond between the α-amino and the γ-carboxylic acid groups of D/L-glutamic acid [1]. Due to its degradability, nontoxicity, good water solubility, and biocompatibility properties, γ-PGA has been applied in agriculture to enhance crop yields. Previous studies have demonstrated that γ-PGA can improve the physicochemical properties of soil. For example, γ-PGA can increase soil moisture, thereby increasing water–fertilizer efficiency and promoting nutrient uptake by plants [2,3]. Moreover, γ-PGA application alters the availability of nitrogen (N), phosphorus (P), and potassium (K) in soil, leading to improved nutrient use efficiency [4,5].

The impact of γ-PGA on soil N cycling has obtained significant attention; it can regulate soil N transformation by altering the soil microbial community composition, particularly the abundance of N-related functional genes [6,7,8]. In addition, γ-PGA application can influence soil urease activity and other critical enzymes—such as sucrase, catalase, and phosphatase—which are intrinsically linked to nutrient cycling and soil fertility [4,9]. Moreover, the application rate of γ-PGA has been shown to significantly influence N fertilizer utilization efficiency [10,11].

Crucially, the molecular weight (Mw) of γ-PGA plays a critical role in determining its applications. Generally, low Mw (<400 kDa) γ-PGA is suitable for pharmaceutical applications, while high Mw (700–1000 kDa) and ultra-high Mw (>1000 kDa) γ-PGAs are used as thickeners or biomaterials [12,13]. Thus, γ-PGAs with different Mws may exhibit distinct functional properties. Recent studies have increasingly focused on the impact of γ-PGA Mw in various applications. For instance, when γ-PGAs with Mws of 700, 1000, 2000, and 3000 kDa were used to modify fish gelatin, the 3000 kDa γ-PGA significantly enhanced the gelling properties [14]. In another study, 732 kDa γ-PGA exhibited better water retention in minced beef paste than γ-PGA of 341 and 1304 kDa [15]. However, most agricultural studies have only examined the effects of γ-PGA with a single Mw. For example, γ-PGA at 1000 kDa has been shown to improve the N use efficiency [16], while γ-PGA at 700 kDa enhanced soil physicochemical properties and winter wheat production [17]. To our knowledge, the comparative effects of γ-PGAs with different Mws on plant growth and soil properties has not been systematically investigated.

Previous studies have shown that γ-PGA can reduce water infiltration and evaporation by increasing soil viscosity [18,19]. Since viscosity of γ-PGA is positively correlated with both Mw and application rate [20], we hypothesized that the γ-PGA with higher Mws or application rates would further reduce soil cumulative infiltration and infiltration rates. Moreover, soil microbial community structure is sensitive to changes in soil moisture content [21]. These changes may subsequently influence nutrient availability and plant growth. Despite these potential linkages, the mechanisms by which γ-PGA Mw regulates soil physicochemical properties and soil microbial communities in plant–soil systems have not been systematically investigated. Therefore, this study evaluates the effects of γ-PGAs with different Mws on soil physicochemical properties, lettuce growth, and soil bacterial community structure. The results show that 0.1% ultra-high Mw γ-PGA is more effective in promoting lettuce growth and improving soil properties. However, the excessive application of ultra-high Mw γ-PGAs inhibited plant growth. These findings can provide insight into the agricultural application of γ-PGA.

## 2. Materials and Methods

### 2.1. γ-PGA, Soil and Chemical Materials

Low Mw (70–100 kDa, purity 92%) and high Mw (700–1100 kDa, purity 92%) γ-PGAs were purchased from Furuida Biotechnology Co., Ltd. (Jinan, Shandong, China). Ultra-high Mw (>3000 kDa) γ-PGA was produced as previously described [22]. The soil used in the pot experiments was obtained from Guiyuxin Agricultural Technology Co., Ltd. (Nanning, China). The soil chemical analysis kits, including those for ammonium N (NH_4_^+^-N), nitrate N (NO_3_^−^-N), available P (AP), available K (AK), acid phosphatase, and neutral phosphatase, were purchased from Solarbio Science & Technology Co., Ltd. (Beijing, China).

### 2.2. Infiltration Soil Column Experiment

The effects of γ-PGA on soil water-holding capacity were evaluated using a soil column infiltration experiment. The initial characteristics of the red soil were as follows: pH (1:2.5 soil/water ratio) 4.9, 25.56 μg/g available N, 10.2 μg/g AP, and 158.7 μg/g AK. The red soil was air-dried and sieved with 20 mesh. Soil columns were constructed using 0.5 cm thick organic glass tubes (8 cm inner diameter, 40 cm height, with 30 cm soil depth), featuring seven lateral openings spaced 5 cm apart. A 2 cm water layer was maintained above the soil surface during infiltration. A Mars bottle (5 cm inner diameter, 0.5 cm wall thickness, 22.5 cm height) made of organic glass was used. Filter paper was placed at the bottom of the columns to prevent soil particle loss and outlet blockage during infiltration.

### 2.3. Design of Pot Experiment

The effects of γ-PGA on plant growth were investigated using pot experiments conducted outdoors at Guangxi University, China (22°50′28.41″ N, 108°17′9.00″ E) from September to November 2022. The initial characteristics of the nutrient soil were as follows: pH (1:2.5 soil/water ratio) 7.53, 449.42 μg/g available N, 64.11 μg/g AP and 217.25 μg/g AK. γ-PGAs with different Mws and concentrations were thoroughly mixed with 2 kg of soil per treatment according to the groups listed in Table 1 and placed in 8 L pots. High-quality seeds of lettuce were chosen and germinated. Seedlings with the same robustness were transferred into pots. Each treatment involved three replicate pots with one seedling planted in each pot. The pots were randomly arranged outdoors and maintained for 56 days.

### 2.4. Assays of Soil Chemical Characteristics and Lettuce Growth

To evaluate the effects of γ-PGA application on soil nutrient availability, enzyme activities, and lettuce growth performance, rhizosphere soil samples were collected on day 56 for chemical and microbial analyses. Soil NH_4_^+^-N, NO_3_^−^-N, AP, and AK contents were determined using the corresponding commercial detection kits based on colorimetric or turbidimetric reactions followed by UV–visible spectrophotometric detection. Specifically, NH_4_^+^-N, NO_3_^−^-N, AP, and AK were quantified using commercial assay kits (BC1510, BC0045, BC2960 and BC3045; Solarbio, Beijing, China) according to the manufacturers’ protocols. The assays were based on the indophenol blue method, nitrosalicylic acid method, molybdenum–antimony colorimetric method, and potassium tetraphenylborate method, respectively. Absorbance was measured using a UV–visible spectrophotometer (UVmin-1240, Shimadzu, Kyoto, Japan), and standard solutions were used for calibration during each measurement.

Soil urease activity was measured using the indophenol blue colorimetric [23]. Catalase activity was determined as previously described [24]. Acid and neutral phosphatase activities were assessed using commercial assay kits (BC0140 and BC0460; Solarbio) according to the manufacturers’ instructions. All soil analyses were performed in triplicate.

The lettuces were harvested on day 56. The growth parameters measured included blade length and width, stem length, and root length. All tests were performed in triplicate.

### 2.5. Analysis of Soil Bacterial Community

Soil bacterial community compositions were analyzed by high-throughput sequencing. Total DNA from soil samples were extracted using the PowerSoil DNA Isolation Kit (MoBio, Carlsbad, CA, USA). The V4–V5 region of the 16S rRNA gene was amplified using primers 515F (5′-GTGCCAGCMGCCGCGG-3′) and 907R (5′-CCGTCAATTCMTTTRAGTTT-3′). Sequencing was performed on an Illumina MiSeq PE300 platform (Illumina, Inc., San Diego, CA, USA). Raw sequence data were deposited in the NCBI database under accession number PRJNA1132897.

The Illumina sequencing-derived data were analyzed online using the Majorbio Cloud Platform (https://www.majorbio.com (accessed on 29 May 2024)). Raw sequences were trimmed via FLASH v1.2.11 and quality controlled via Fastp v0.19.6. Sequences with similarity above 97% were assigned to operational taxonomic units (OTUs) using UPARSE analysis with the SILVA database (https://www.arb-silva.de (accessed on 29 May 2024)). Diversity indices (Chao1, Shannon, Simpson, ACE, and coverage) were calculated using Mothur v.1.30.2. Principal co-ordinates analysis (PCoA) was conducted via QIIME v1.9.1. Distance-based redundancy analysis (dbRDA) was conducted using the vegan package (v2.4.3) in R.

### 2.6. Statistical Analysis

Statistical analyses were conducted using SPSSAU (Online platform, www.spssau.com (accessed on 30 May 2024)). The differences among treatments were evaluated using one-way analysis of variance (ANOVA). When significant differences were detected, multiple comparisons were conducted using the Waller–Duncan multiple range test at a significance level of *p* < 0.05. Figures were prepared using Origin 2021 (OriginLab Corporation, Northampton, MA, USA).

## 3. Results

### 3.1. Effect of γ-PGA on Red Soil Infiltration

**Figure 1:**
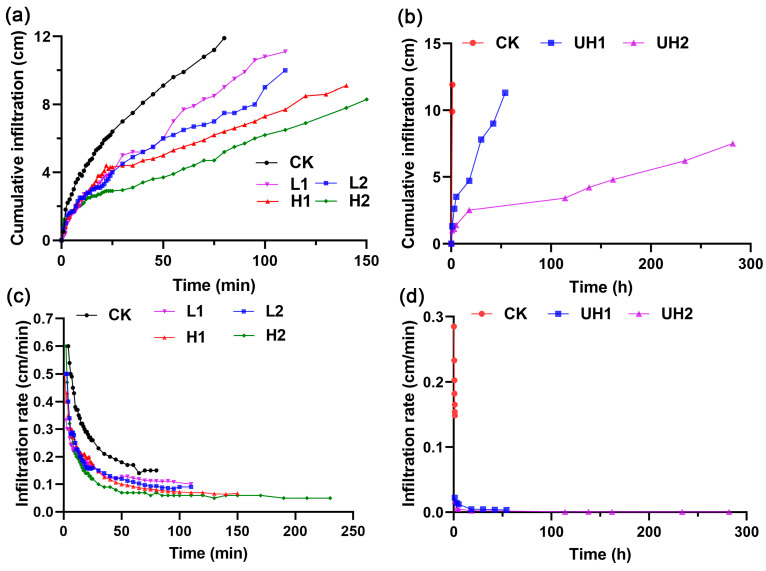
Effects of γ-PGA on soil infiltration characteristics: (**a**,**b**) cumulative infiltration; (**c**,**d**) infiltration rate.

The cumulative infiltration of red soil treated with γ-PGA was significantly reduced compared to the control (CK, 9.9 cm) after 60 min (Figure 1a,b). The values were as follows: 7.7 cm (L1), 6.5 cm (L2), 5.5 cm (H1), 4.2 cm (H2), 1.3 cm (UH1), and 1.0 cm (UH2). These values represented reductions of 22.22% (L1), 34.34% (L2), 44.44% (H1), 57.58% (H2), 86.87% (UH1), and 89.9% (UH2) relative to the CK. The data indicated that cumulative infiltration decreased with increasing γ-PGA Mw and application rate. In particular, ultra-high Mw γ-PGA substantially inhibited soil infiltration. As shown in Figure 1c, the infiltration rates for all γ-PGA treatments were lower than those of the CK. The infiltration rates stabilized at approximately 65 min for L1 (0.119 cm/min), L2 (0.103 cm/min), H1 (0.087 cm/min), and H2 (0.070 cm/min), representing reductions of 20.67%, 31.33%, 42%, and 53.33%, respectively, compared with the CK. Notably, the infiltration rates of the ultra-high Mw treatments (UH1 and UH2) required significantly more time to stabilize—18 h for UH1 and 114 h for UH2 (Figure 1d). These results suggested that γ-PGA application markedly slowed water movement in soil, likely due to the increased solution viscosity and water-binding capacity. Such changes may contribute to improved soil water retention.

### 3.2. Lettuce Traits Analysis

**Figure 2:**
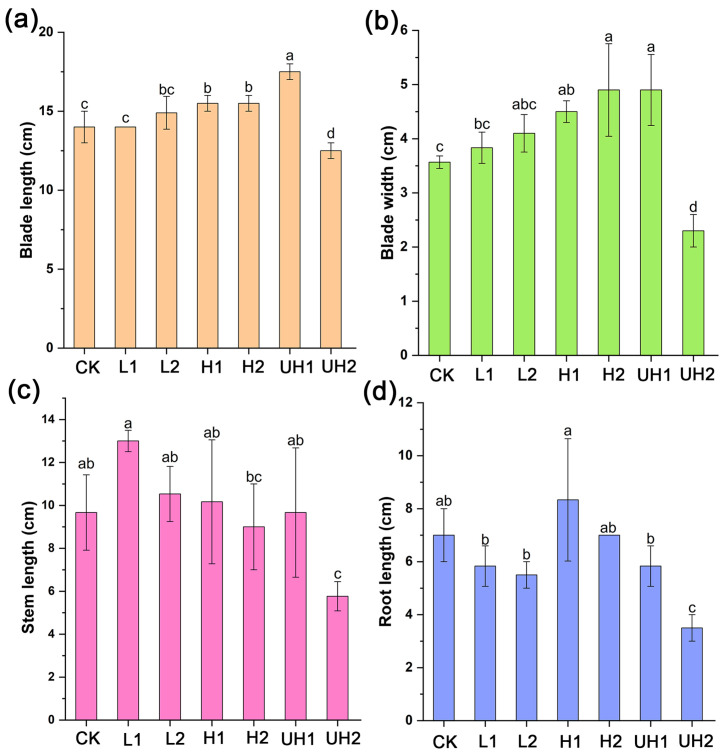
Effect of γ-PGA on lettuce growth: (**a**) blade length; (**b**) blade width, (**c**) stem length; (**d**) root length. Error bars represent standard deviations of triplicate measurements. Different letters (a–d) are annotated on graphs to indicate statistical significance among treatments at *p* < 0.05.

The analysis of lettuce traits indicated that their blade length and width roughly increased with the Mw and application rate of γ-PGA, except for the UH2 treatment (Figure 2a,b). Compared with the CK, the blade length and width of the lettuce in the UH1 treatment increased by 25% and 37.25%, respectively. Notably, the lettuce size in the UH2 treatment was lower than in all other γ-PGA treatments and even the control, indicating that a high rate of ultra-high Mw γ-PGA may inhibit lettuce growth. Although lettuce stem lengths in the L1, L2, H1, and UH1 treatments were greater than the CK, the differences were not statistically significant (Figure 2c). There was no significant difference in the lettuce root length between γ-PGA treatments and the control except for the UH2 treatment (Figure 2d). In addition, the application rate (0.1% vs. 0.2%) had no significant effect on lettuce growth under low and high Mw γ-PGA treatments. Overall, these results suggested that γ-PGA can promote lettuce growth, and this effect appeared to be positively correlated with the γ-PGA Mw. However, for ultra-high Mw γ-PGA, the application dosage should be considered, as excessive application may negatively affect lettuce growth in soil.

### 3.3. Effect of γ-PGA on Soil NH_4_^+^-N, NO_3_^−^-N, AP, and AK Contents

**Figure 3:**
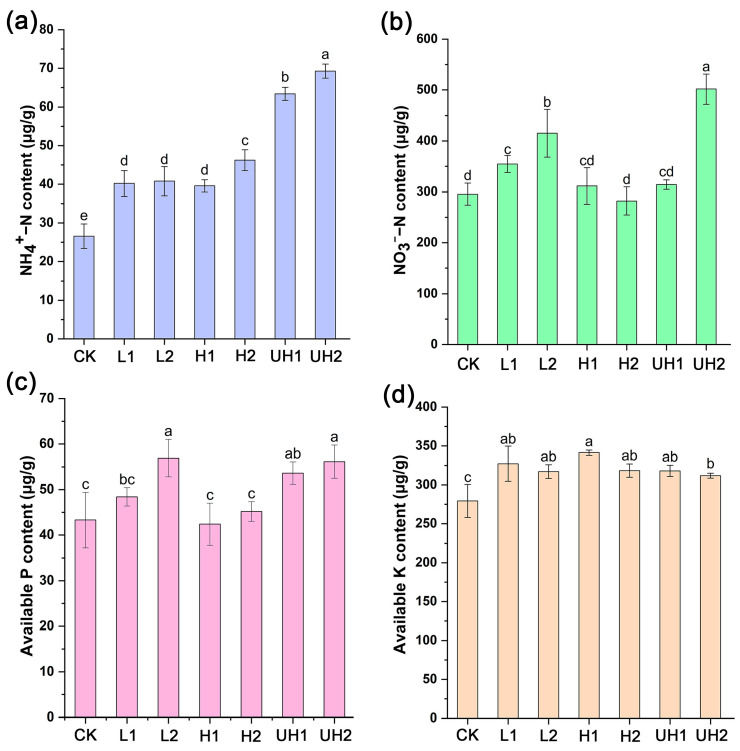
Effects of γ-PGA on soil NH_4_^+^-N (**a**), NO_3_^−^-N (**b**), AP (**c**), and AK (**d**) contents. Error bars represent standard deviations of triplicate measurements. Different letters (a–e) are annotated on graphs to indicate statistical significance among treatments at *p* < 0.05.

The effects of γ-PGA on soil chemical properties were further assayed. The results showed that the soil NH_4_^+^-N content significantly increased in all γ-PGA treatments compared to the CK (Figure 3a). Notably, the UH1 and UH2 treatments exhibited greater increases, with NH_4_^+^-N levels elevated by 138.52% and 160.73%, respectively. Although no significant difference was observed between L1 and L2, soils receiving the higher application rate of γ-PGA generally showed a higher NH_4_^+^-N content than those receiving the lower rate. These findings indicated that γ-PGA application enhanced soil NH_4_^+^-N content, with ultra-high Mw γ-PGA having the most pronounced effect.

Regarding soil NO_3_^−^-N content, significant increases were observed in the L1, L2, and UH2 treatments relative to the CK, with respective increases of 20%, 40.56%, and 69.84% (Figure 3b). No significant differences were detected in the other γ-PGA treatments compared to the CK. With respect to the application rate, NO_3_^−^-N content under the high-rate treatment was significantly greater than that under the low-rate treatment for both low and ultra-high Mw γ-PGAs. This indicated that the influence of γ-PGA on NO_3_^−^-N content was dependent on the specific Mw and application rate combinations.

The measurement of soil AP content revealed that the γ-PGA application significantly increased AP content in the L1, L2, UH1, and UH2 treatments, with respective increases of 11.78%, 31.4%, 23.85%, and 14.11% (Figure 3c). A high application rate of γ-PGA with specific Mws resulted in greater AP contents compared with a low rate. However, no significant change was observed in the H1 and H2 treatments, suggesting that high Mw γ-PGAs may have limited effects on AP content.

As shown in Figure 3d, γ-PGA application also significantly increased soil AK content across all treatments compared to the CK, with increases of 17.13% (L1), 13.53% (L2), 22.24% (H1), 13.99% (H2), 13.86% (UH1), and 11.68% (UH2). Despite the consistent improvement in AK content, there were no statistically significant differences between the γ-PGA Mw and application rate.

These findings indicated that γ-PGA can enhance soil nutrient levels, particularly NH_4_^+^-N and AK. While increases in NH_4_^+^-N were strongly associated with ultra-high Mw γ-PGA, improvements in AP and NO_3_^−^-N contents appeared to vary depending on the Mw and application rate. In contrast, AK content was generally improved regardless of γ-PGA Mw or application rate.

### 3.4. Soil Enzyme Activities

**Figure 4:**
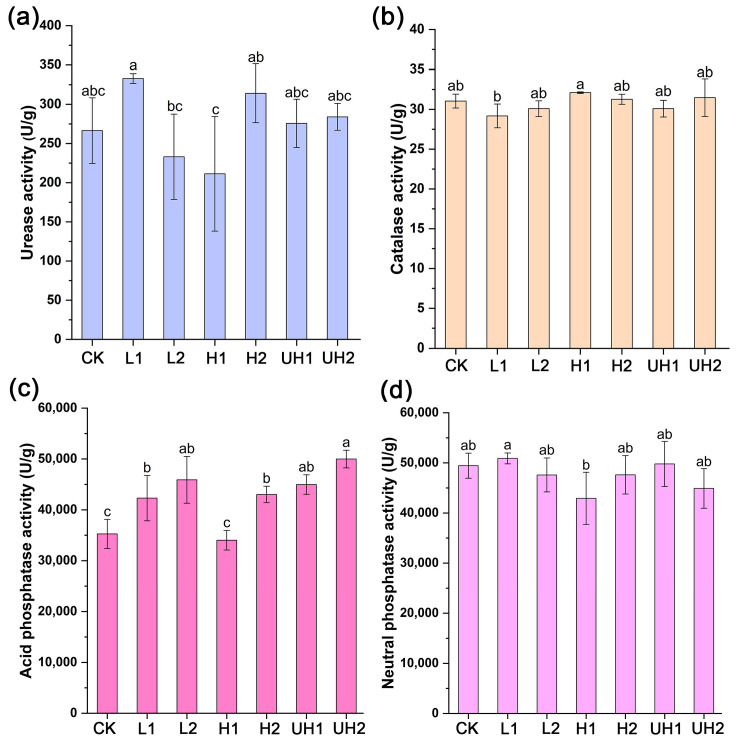
Effects of γ-PGA on soil enzyme activities: (**a**) urease; (**b**) catalase; (**c**) acid phosphatase; (**d**) neutral phosphatase. Error bars represent standard deviations of triplicate measurements. Different letters (a–c) are annotated on graphs to indicate statistical significance among treatments at *p* < 0.05.

In this study, soil urease, catalase, acid phosphatase, and neutral phosphatase activities were evaluated to assess the impact of γ-PGA application. As shown in Figure 4a, although the soil urease activities in the L1, H2, UH1, and UH2 treatments were higher than that in the control, the differences were not statistically significant. With respect to the application rate, significant differences were observed between low- and high-rate treatments for low and high Mw γ-PGAs, whereas no significant differences were detected between rates under ultra-high Mw γ-PGA treatments.

Similarly, no significant differences in catalase activity were observed between γ-PGA-treated soils and the control (Figure 4b), nor were there significant effects due to the application rate. These results indicated that γ-PGA had little to no effect on soil urease and catalase activities.

In contrast, soil acid phosphatase activity was significantly enhanced in the L1, L2, H1, UH1, and UH2 treatments compared to the CK, increasing by 19.98%, 30.18%, 22.03%, 27.55%, and 41.78%, respectively (Figure 4c). However, activity was slightly reduced in the H2 treatment. A high application rate tended to enhance acid phosphatase activity compared with a low rate, although this effect was statistically significant only for high Mw γ-PGA treatments. These results indicated that γ-PGA with low and ultra-high Mws, particularly when applied at higher rates, can improve the soil acid phosphatase activity.

Neutral phosphatase activity was not significantly affected by γ-PGA application or by the application rate (Figure 4d), indicating that γ-PGA had no significant impact on this enzyme.

In summary, among the four soil enzymes tested, only acid phosphatase activity was significantly affected by γ-PGA application, particularly in response to low and ultra-high Mw γ-PGA treatments.

### 3.5. Soil Bacterial Diversity

To investigate the effects of γ-PGA application on the soil microbial community, 16S rRNA sequencing was conducted on soil samples from all treatments and the CK. The sequencing coverage for all samples exceeded 0.98 (Table 2), indicating a sufficient sequencing depth to accurately represent the bacterial community structures. Alpha diversity analysis revealed that most γ-PGA treatments led to a reduction in bacterial diversity compared to the CK, as indicated by lower Shannon and Simpson indices. Similarly, bacterial richness, assessed by ACE and Chao1 indices, also decreased in the γ-PGA-treated groups. However, no statistically significant differences in bacterial diversity or richness were observed between the L2, UH1, and UH2 treatments and the control. In addition, while significant differences in alpha diversity and bacterial richness were detected between the low and high application rates in low Mw γ-PGA treatments, no significant rate-dependent effects were observed under high or ultra-high Mw γ-PGA treatments. These results suggested that γ-PGA with a low Mw at a higher concentration and an ultra-high Mw application had minimal impact on soil microbial diversity and richness.

**Figure 5:**
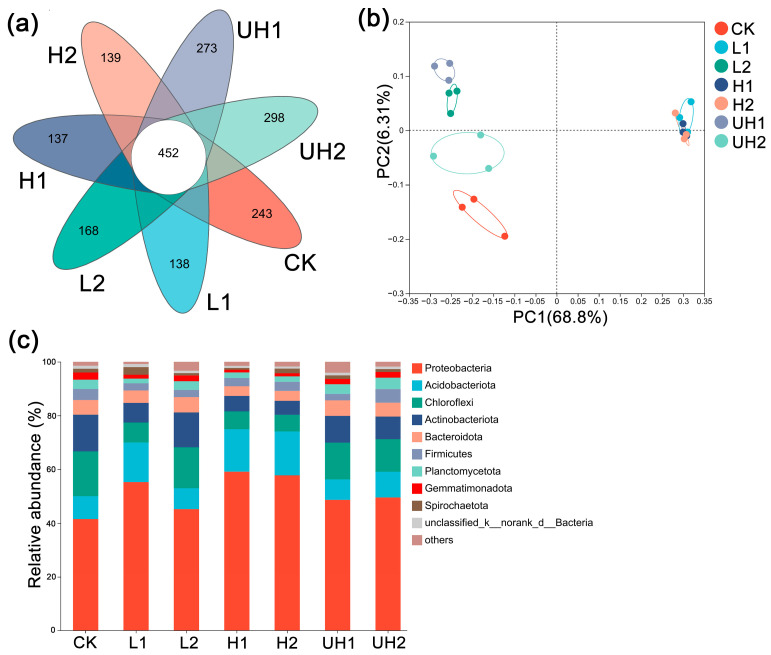
Venn diagram of bacterial OTU numbers (**a**), PCoA of bacteria community (**b**), and relative abundances of microorganism taxonomic groups (**c**) in different treatments.

Venn diagram analysis demonstrated that a substantial number of OTUs were shared across all treatments (Figure 5a), suggesting that γ-PGA addition did not markedly alter the dominant microbial taxa. Although both low and high Mw γ-PGA treatments reduced overall bacterial diversity, ultra-high Mw γ-PGA treatments exhibited a greater number of unique OTUs than the CK and other treatments. This indicated that while the dominant community structure remained largely unaffected, γ-PGA, particularly at ultra-high Mws, may influence the presence of specific microbial populations.

### 3.6. Similarity Analysis of Soil Bacterial Communities

PCoA based on Bray–Curtis distance at the OTU level was conducted to evaluate the differences in soil bacterial community composition among treatments. The first and second principal coordinates explained 68.8% and 6.31% of the total variance in bacterial community composition, respectively (Figure 5b). Notably, the L1, H1, and H2 treatments clustered closely together, suggesting that γ-PGA with a low Mw at a low rate and a high Mw resulted in similar microbial community structures. These results suggested that γ-PGA application significantly affected the bacterial communities.

### 3.7. Soil Bacterial Community Composition

As shown in Figure 5c, *Proteobacteria* (41.19–59.11%), *Acidobacteriota* (6.45–16.33%), *Chloroflexi* (6.19–17.10%), and *Actinobacteriota* (5.19–13.67%) were the predominant phyla found in all soil samples. Among these, *Proteobacteria* exhibited the highest relative abundance in all treatments. Notably, γ-PGA application increased the relative abundance of *Proteobacteria*, suggesting that γ-PGA promoted the proliferation of this phylum. The relative abundance of *Acidobacteriota* increased in the L1, H1, and H2 treatments, when compared to the control, by 6.25%, 7.32%, and 7.84%, respectively. In contrast, *Chloroflexi* abundance decreased in most γ-PGA treatments, except for L2. Furthermore, *Actinobacteriota* showed a marked decline in relative abundance, particularly in the L1, H1, and H2 treatments. These results indicated that γ-PGA application altered the composition of the soil bacterial community, more specifically, primarily by enhancing the relative abundance of *Proteobacteria* while reducing the relative abundance of *Actinobacteriota*.

### 3.8. Relationship Between the Bacterial Community and Soil Biochemical Factors

**Figure 6:**
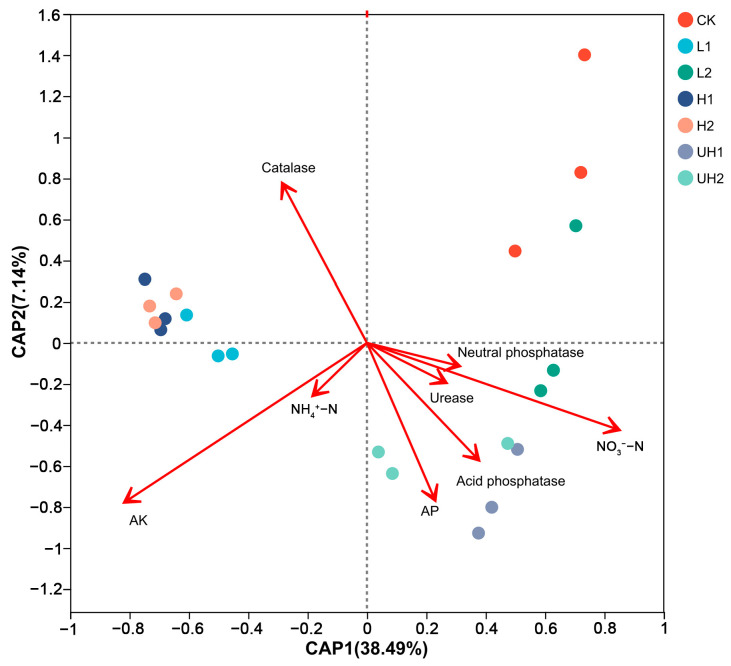
Correlations between bacterial communities and soil biochemical factors at phylum level.

dbRDA was performed to explore the relationship between soil biochemical factors and the bacterial community structure. The results indicated that the bacterial community composition was strongly influenced by NO_3_^−^-N, AK, and catalase activity (Figure 6). For the first axis, the most important biochemical factors were neutral phosphatase and NO_3_^−^-N; for the second axis, the important biochemical factor was AP. Notably, AP was positively associated with the activities of neutral phosphatase and acid phosphatase. In contrast, AK content exhibited a negative correlation with the bacterial community structure (r^2^ = 0.60, *p* = 0.001), suggesting that higher AK levels may suppress certain microbial populations.

## 4. Discussion

### 4.1. The Decrease in Soil Infiltration Rates by γ-PGA Application

Red soil is widely distributed in tropical and subtropical areas of South China. Its characteristic red or yellow color results from the enrichment of iron and aluminum oxides, giving the soil its name. However, red soil generally exhibits low water-holding capacity [25,26]. Therefore, red soil was selected to evaluate the effect of γ-PGA on soil infiltration. In this study, γ-PGA application reduced the infiltration rates of red soil. This was likely due to γ-PGA’s ability to absorb water and form hydrogels, which contributed to the stabilization of water-stable aggregates [2]. However, ultra-high Mw γ-PGA resulted in a more pronounced decrease in infiltration rate compared to low and high Mw γ-PGAs. This effect may be attributed to its higher viscosity [20], which likely increased the viscosity of soil liquid phase, thereby reducing the infiltration rate of the soil [18]. Given the inherent high acidity and poor nutrient content of red soil [27], it may not be ideal for evaluating plant growth. Consequently, nutrient soil was selected for subsequent experiments to better assess the impact of γ-PGA on crop growth.

### 4.2. Influence of γ-PGAs with Different Mws on Lettuce Growth

Previous studies have demonstrated that γ-PGA application can promote plant growth. However, the influence of its Mw on plant growth remains unclear. Our findings revealed that γ-PGAs with varying Mws enhanced the growth of lettuce. Traits analysis showed that lettuce blade length and width increased significantly under γ-PGA treatments compared to the control (Figure 2), and this effect exhibited a generally positive correlation with both the Mw and application rate of γ-PGA in soil. This was because of γ-PGA’s ability to enhance soil water retention, thereby improving nutrient availability and uptake which ultimately promoted plant growth [4,18,28]. Moreover, γ-PGA application has been reported to improve N use efficiency and enhance biological N fixation [10,29].

Although γ-PGA application did not significantly increase soil urease activity, soil NH_4_^+^-N content in the γ-PGA treatments were higher than the CK (Figure 3). This increase may be explained by multiple mechanisms: First, γ-PGA contains abundant carboxyl groups that can adsorb NH_4_^+^ through electrostatic interactions [30], thereby reducing NH_4_^+^ loss. Notably, soil NH_4_^+^-N content in UH1 and UH2 treatments were significantly higher than the other γ-PGA treatments. We speculated that ultra-high Mw γ-PGAs may have greater NH_4_^+^ adsorption capacity. Second, the enhanced soil moisture by γ-PGA may limit oxygen diffusion and suppress nitrification, leading to reduced conversion of NH_4_^+^-N to NO_3_^−^-N [31,32]. Additionally, γ-PGA can be degraded into soil N fertilizer by microorganisms [33]. Therefore, γ-PGA may enhance the capacity of N fixation. Soil AP contents were also elevated in the γ-PGA treatments, which may be associated with enhanced acid phosphatase activity (Figure 4). In addition, γ-PGA applications significantly increased soil AK content. Thus, these improvements in soil nutrient status likely contributed to the enhanced lettuce growth observed in most γ-PGA treatments.

However, the application of 0.2% ultra-high Mw γ-PGA inhibited lettuce growth. This inhibitory effect was likely due to the increased viscosity associated with the high Mw and concentration of the γ-PGA, which reduced the soil aeration state and thereby inhibited the crop root growth [10,20,34]. Our infiltration experiments supported this, as UH2 treatments exhibited the slowest infiltration rates. When ultra-high Mw γ-PGA was applied at a high rate in the soil for a long time, this could have affected the osmotic pressure of the crop root system, resulting in the death of the crop roots [35]. Therefore, the appropriate concentrations of γ-PGA for agricultural application require further systematic investigation.

### 4.3. Correlations Between Soil Bacterial Community and Biochemical Properties

Soil biochemical properties have been closely linked to the bacterial community [36,37]. In this study, although the application of γ-PGA appeared to reduce bacterial diversity, there were no statistically significant differences between the CK and the UH1 and UH2 treatments, indicating that the ultra-high Mw γ-PGAs did not significantly affect overall bacterial diversity. Venn diagram analysis showed a higher number of unique OTUs in the ultra-high Mw γ-PGA treatments (Figure 5), suggesting that these may support distinct bacterial populations or promote bacterial growth. At the phylum level, *Proteobacteria*, *Acidobacteriota*, *Actinobacteriota*, and *Chloroflexi* were dominant across all treatments, which was consistent with previous studies [3,5]. γ-PGA application increased the relative abundance of *Proteobacteria* in soil. Most members of *Proteobacteria* were important for the global carbon, N, and sulfur cycles in soil [38,39]. The relative abundance of *Acidobacteriota* in the L1, H1, and H2 treatments was higher than that in the control. *Proteobacteria* and *Actinobacteriota* are related to the urease microbial community in soil [40], and *Acidobacteria*-related bacteria can play an important role in nutrient cycling in soil environments [5]. Their increased abundance could suggest that application of γ-PGA with specific Mws may enhance microbial processes associated with nutrients. *Chloroflexi* are involved in the degradation of organic matter and the carbon cycle [41,42]. However, the relative abundance of *Chloroflexi* decreased in most γ-PGA treatments. These results suggested that the application of γ-PGA may mainly promote the growth of N-cycling-related bacterium in the soil. The dbRDA results further confirmed that soil nutrient availability and enzymatic activities were key drivers shaping bacterial community composition. Compared with previous studies, this study provided a systematic comparison of low, high, and ultra-high Mw γ-PGAs within the same soil–plant system. At a low application rate, the ultra-high Mw γ-PGA was more effective in promoting lettuce growth and improving soil properties. Relative to the CK, lettuce grown under the UH1 treatment exhibited a 25% increase in blade length and a 37.25% increase in blade width, while cumulative infiltration decreased by 86.87% and soil NH_4_^+^-N increased by 138.52%. However, excessive application of ultra-high Mw γ-PGAs inhibited plant growth, highlighting the necessity of optimizing application rates according to their Mw.

## 5. Conclusions

In summary, γ-PGAs with different Mws significantly influenced soil properties. As the Mw and application rate of γ-PGA increased, the soil water-holding capacity may have improved. γ-PGA can promote lettuce growth, and this effect was positively correlated with γ-PGA Mws and application rates. However, the application of a high rate of ultra-high Mw γ-PGA may negatively affect lettuce growth, likely due to restricted root development caused by excessive water retention. Furthermore, γ-PGA treatments altered the soil bacterial community structure, potentially enhancing nutrient availability. Overall, γ-PGA improved plant performance through its effects on soil physicochemical and microbial properties. These findings can offer valuable insights into the agricultural application of γ-PGA and underscore the importance of selecting appropriate Mws and application rates for optimal plant growth.

## Figures and Tables

**Table 1:** γ-PGA treatments in pot experiments.

Treatments	CK	L1	L2	H1	H2	UH1	UH2
γ-PGA rate (w:w)	0	0.1%	0.2%	0.1%	0.2%	0.1%	0.2%

CK: control; L1 and L2: low Mw γ-PGA; H1 and H2: high Mw γ-PGA; UH1 and UH2: ultra-high Mw γ-PGA.

**Table 2:** Analysis of bacterial diversity indices in soil samples.

Treatments	Shannon	Simpson	ACE	Chao1	Coverage
CK	5.289 a	0.012 c	1557 a	1505 a	0.985
L1	4.394 b	0.034 a	995 b	978 b	0.99
L2	5.213 a	0.014 c	1552 a	1430 a	0.986
H1	4.45 b	0.033 ab	1207 b	1115 b	0.989
H2	4.517 b	0.029 b	1111 b	1085 b	0.989
UH1	5.289 a	0.013 c	1523 a	1489 a	0.985
UH2	5.191 a	0.015 c	1529 a	1458 a	0.985

Different letters (a–c) are annotated on graphs to indicate statistical significance among treatments at *p* < 0.05.

## Data Availability

All data are available in the main text. The raw sequencing data have been uploaded to the NCBI database (BioProject ID: PRJNA1132897).

## Abbreviations

The following abbreviations are used in this manuscript:

γ-PGA	Poly-γ-Glutamic Acid
Mw	Molecular Weight
N	Nitrogen
P	Phosphorus
K	Potassium
Ammonium N	NH_4_^+^-N
Nitrate N	NO_3_^−^-N
AP	Available P
AN	Available N
AK	Available K
OTUs	Operational Taxonomic Units
PCoA	Principal Co-Ordinates Analysis
LDA	Linear Discriminant Analysis
LEfSe	LDA Effect Size
dbRDA	Distance-Based Redundancy Analysis
